# Short-Term Supplementation With Fermented Red Clover Extract Reduces Vascular Inflammation in Early Post-menopausal Women

**DOI:** 10.3389/fcvm.2022.826959

**Published:** 2022-02-10

**Authors:** Kate A. Wickham, Line B. Nørregaard, Mikkel Oxfeldt, Stephen S. Cheung, Lasse Gliemann, Mette Hansen, Ylva Hellsten

**Affiliations:** ^1^Department of Nutrition, Exercise and Sports, University of Copenhagen, Copenhagen, Denmark; ^2^Environmental Ergonomics Lab, Faculty of Applied Health Sciences, Brock University, St. Catharines, ON, Canada; ^3^Department of Public Health, Aarhus University, Aarhus, Denmark

**Keywords:** fermented red clover, phytoestrogen, isoflavones, post-menopausal women, supplementation, cardiovascular health, vascular inflammation, skeletal muscle microcirculation

## Abstract

The decline in estrogen at menopause poses a critical challenge to cardiovascular and metabolic health. Recently, a growing interest in the role of phytoestrogens, with a particular focus on isoflavones, has emerged as they can bind to estrogen receptors and may mimic the roles of endogenous estrogen. Fermented red clover extract (RC) contains isoflavones with superior bioavailability compared to non-fermented isoflavones, however little is known regarding the impact of isoflavones on cardiovascular and metabolic health. We assessed markers of vascular health in plasma and skeletal muscle samples obtained from healthy but sedentary early post-menopausal women (*n* = 10; 54 ± 4 years) following 2 weeks of twice daily treatment with placebo (PLA) or RC (60 mg isoflavones per day). The two interventions were administered using a randomized, double-blind, crossover design with a two-week washout period. Plasma samples were utilized for assessment of markers of vascular inflammation. There was a statistically significant reduction (~5.4%) in vascular cell adhesion molecule 1 (VCAM-1) following 2 weeks of RC supplementation compared to PLA (*p* = 0.03). In contrast, there was no effect of RC supplementation compared to PLA on skeletal muscle estrogen receptor content and enzymes related to vascular function, and angiogenesis. Supplementation with RC reduces vascular inflammation in early post-menopausal women and future studies should address the long-term impact of daily supplementation with RC after menopause.

## Introduction

Existing evidence clearly highlights that the loss of estrogen associated with menopause poses a critical challenge to cardiovascular and metabolic health (1, 2). Notably, compared to age-matched men, post-menopausal women have a similar, if not greater, risk of developing cardiovascular and metabolic disease (3–6). In pre-menopausal women, estrogen plays a cardioprotective role through the activation of several signaling cascades, which lead to the promotion of vasodilation and angiogenesis as well as the reduction in oxidative stress and fibrosis (7). Additionally, the biochemical structure of estrogen possesses direct anti-inflammatory characteristics, where the hydroxyl group of its aromatic ring can quench superoxide ions (8). Accordingly, the withdrawal of estrogen production associated with menopause has been shown to increase oxidative stress and subsequently induce negative effects on cardiovascular health including increased sympathetic tone, endothelial dysfunction, vascular inflammation, and increased blood pressure (9, 10). Moreover, endothelial dysfunction and subsequent alterations in vascular tone and blood flow have significant implications for peripheral tissue metabolism, whereby skeletal muscle angiogenesis can become impaired (11, 12).

Hormone replacement therapy (HRT) has been a leading therapeutic intervention for the maintenance of cardiovascular and metabolic health in post-menopausal women (13). However, there is mounting concern regarding the efficacy and safety of this treatment (14). Specifically, it appears that HRT may not be an effective strategy for mitigating cardiovascular disease development if started late into menopause (15, 16). Consequently, there is an urgent call for novel, safe, and efficacious therapeutic interventions that can promote cardiovascular and metabolic health in post-menopausal women. Recently, a growing interest in the role of phytoestrogens, with a particular focus on isoflavones, has emerged. Isoflavones are predominantly derived from legumes and can bind to both estrogen receptor alpha (ERα) and estrogen receptor beta (ERβ), thereby potentially mimicking the roles of endogenous estrogen (17, 18). Interestingly, although 17β-estradiol binds with equal affinity to ERα and ERβ (19), phytoestrogens appear to have a higher affinity for ERβ (17). Epidemiological data suggests that women ingesting large amounts of phytoestrogens are less likely to develop cardiovascular disease as well as breast and uterine cancer (20, 21). Specifically, long-term (≥ 6 weeks) soy isoflavone supplementation has been linked to increased nitric oxide (NO) bioavailability (22) and prostacyclin (PGI_2_) release (23) as well as reduced endothelin-1 levels (22) in post-menopausal women. Moreover, a growing body of evidence suggests that long-term soy isoflavone supplementation can improve markers on vascular inflammation in post-menopausal women (24–26). Importantly, these findings translate to an improvement in vascular function (27).

Red clover (*trifolium pratense*), a plant known for its high phytoestrogen content, is beginning to gain traction as a novel therapeutic intervention for post-menopausal women as it is rich in the isoflavones biochanin A and formonentin (28). Though limited, previous research has shown that supplementation with red clover extract can confer positive effects on vascular function in post-menopausal women (29, 30). However, the findings regarding blood markers of cardiovascular health are currently unclear. Some evidence suggests improvements in antioxidant and vasorelaxant properties (31) as well as atherogenic adhesion molecules (32), while others demonstrate no effect of red clover extract on inflammatory markers (33) or coagulation factors (34). A key determinant of nutraceutical efficacy is the bioavailability of the supplement (35). In recent years, advances in food science and nutraceutical biochemistry have significantly improved the bioavailability, and therefore, the therapeutic potential of red clover isoflavones *via* the fermentation process, which converts isoflavone glycosides to aglycones (33). Therefore, fermented red clover extract (RC) may be a safe, easily accessible, and highly efficacious therapeutic intervention to promote cardiovascular and metabolic health as well as bone health (33) and the maintenance of muscle mass and function (36) in post-menopausal women. However, to date, no study has evaluated the cardiovascular benefits in a cohort of post-menopausal women. Therefore, we aimed to determine whether 2 weeks of supplementation with RC could positively influence markers of vascular health in blood and skeletal muscle in early post-menopausal women utilizing a crossover design. We hypothesized that short-term supplementation with RC would significantly improve markers of vascular health in the blood and skeletal muscle of early post-menopausal women.

## Materials and Methods

### Ethical Approval

The human muscle biopsies and blood samples included in the present study originated from a published study conducted at the Department of Public Health, Aarhus University, Denmark (36). The study was in accordance with the Declaration of Helsinki and was approved by the Central Denmark Region Committees on Health Research Ethics (1-10-72-212-19) and registered at Clinical.trials.gov (ID: NCT04154206). All the participants provided written informed consent to participate after they were fully informed about the project as well as the associated risks and discomforts.

### Participants

Ten healthy but sedentary, early post-menopausal women were recruited to participate in this study (age: 54 ± 4 years; height: 168 ± 6 cm; weight: 70 ± 8 kg) *via* posters displayed in the local area and on social media. Women were defined as early post-menopausal if they had not menstruated for at least 6 months, but it had been <5 years since their last menstrual bleeding (8–33 months since last menstruation). Participants were excluded from the study if they met any of the following criteria: body mass index (BMI) > 30, regularly participated in ≥ 3 h of training per week (except for bike transport; <70 km per week), use of hormone replacement therapy or isoflavone supplements, ovariectomy, cardiovascular and metabolic diseases, as well as injuries or use of medication affecting skeletal muscle.

### Study Design

**Figure 1:**
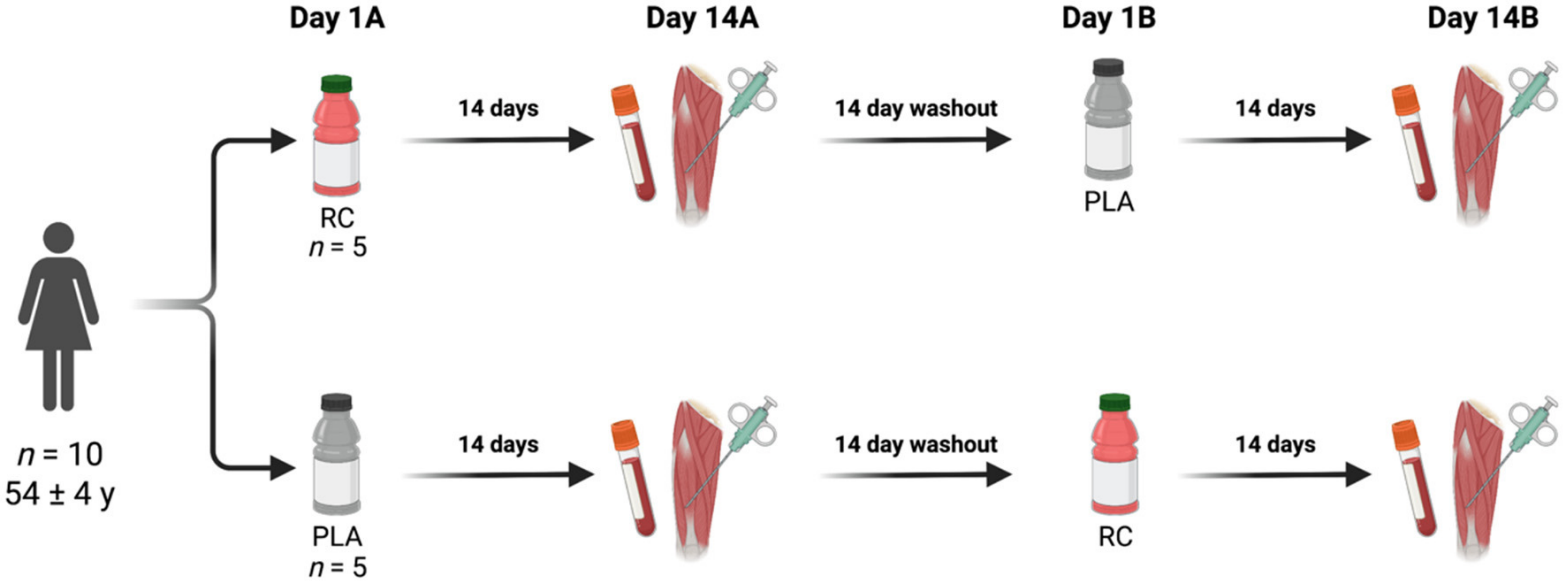
Study design overview. Created with BioRender.com.

Using a double-blind, randomized, crossover design, participants completed two different 14-day intervention periods: fermented red clover extract (RC) and placebo (PLA). The intervention periods were separated by a two-week washout period (14 ± 0 days). On the final day (Day 14) of each experimental period, the participants arrived at the laboratory after an overnight fast. Resting blood samples were obtained from the antecubital vein, where 3.5 mL was collected in a lithium heparin-coated tube. A muscle biopsy was obtained from the middle of the m. vastus lateralis using a Bergström needle with suction under local anesthesia (Figure 1). All muscle samples were immediately frozen in liquid nitrogen and stored at −80°C until further analysis.

### Experimental Drinks

The RC is a commercially available product produced by Herrens Mark Aps (Nørre Aaby, Denmark), and it is comprised of juice from pressed red clover plants mixed with probiotic lactic acid bacteria to facilitate cold fermentation. These bacteria promote the conversion of isoflavone glucosides to aglycones, which improves the bioavailability of the RC. The research team added stevia and natural sugar-free raspberry flavoring to the RC to mask the flavor and appearance of the product. The PLA drink consisted of water with added food coloring as well as stevia and sugar-free raspberry flavoring to match the RC product. The two drinks were sealed and packaged in identical boxes labeled “A” or “B” to ensure adequate blinding of the participants and members of the research team.

The RC and PLA drinks were provided to the participants on the first day of each intervention period. Participants were instructed to consume a daily dose of 120 mL, distributed as 60 mL in the morning and 60 mL in the evening. This dosing regime delivered a minimum of 60 mg isoflavones per day and has been shown to significantly elevate plasma isoflavone levels (37). At the end of the 14-day intervention period, the participants returned the empty containers to the research team as a measure of compliance, which was 100%.

### Blood Analysis

Venous blood was centrifuged immediately (10 min at 1,300 rpm and 5°C). Aliquoted plasma was stored at −80°C until later analysis. Plasma levels of inflammatory markers associated with vascular damage [vascular cell adhesion molecule 1 (VCAM-1), intracellular adhesion molecule 1 (ICAM-1), serum amyloid A (SAA), and C-reactive protein (CRP)] were measured in accordance with the manufacturer's instructions *via* an electrochemiluminescent assay kit (V-PLEX Vascular Injury Panel 2, Meso Scale Diagnostics, USA). Plasma markers are presented for 9 subjects due to difficulties collecting blood from one participant.

### Western Blotting

Approximately 20–50 mg of skeletal muscle was used to determine protein content of estrogen and estrogen-related receptors as well as microvascular enzymes and angiogenic proteins *via* western blot analysis as previously described (38). Briefly, the muscle samples were freeze dried and dissected free of fat, blood, and connective tissue. The samples were then homogenized in a fresh batch of buffer (10% glycerol, 20 mM sodium pyrophosphate, 150 mM NaCl, 50 mM HEPES (pH 7.5), 1% NP-40, 20 mM β-glycerophosphate, 2 mM Na_3_VO_4_, 10 mM NaF, 2 mM PMSF, 1 mM EDTA (pH 8.0), 1 mM EGTA (pH 8.0), 10 μg/mL aprotinin, 10 μg/mL leupeptin, 3 mM benzamidine) twice for 30 s each (Qiagen Tissuelyser II; Retsch, Haan, Germany). After rotation end-over-end for 1 h at 4°C, the suspension was centrifuged, and the lysate was collected for analysis. The concentration of total proteins in the lysate was determined in triplicate *via* BCA protein assay (Pierce Biotechnology Inc., Rockwood, IL, USA). All samples were loaded in duplicates and samples from the same subject were always loaded on the same gel. TGX stain-free gels were used as loading controls, whereby equal protein loading was confirmed by total protein determination from the stain-free image. After gel electrophoresis, proteins were semi-dry transferred to a polyvinylidene difluoride (PVDF) membrane (Immobilon Transfer Membrane, Millipore, MA, USA), which was blocked before overnight incubation with primary antibodies for estrogen receptor alpha (ERα; cs 8644; 1:1,000), estrogen receptor beta (ERβ; mab 7106; 1:500), estrogen-related receptor alpha (ERRα; ab76228; 1:750), fetal liver kinase 1 (VEGFR2, Flk-1; sc 393163; 1:300), cluster of differentiation 31 (CD31; af 806; 1:250), superoxide dismutase 2 (SOD2; Millipore 06-984; 1:5000), endothelial nitric oxide synthase (eNOS; ab76198; 1:1,000), and prostacyclin synthase (PGI_2_S; ab23668; 1:300) (Table 1). Thereafter, membranes were washed for 5 min in Tris-buffered saline Tween (TBST) before incubation with secondary horseradish peroxidase (HRP) conjugated antibody for 1 h. Then, the membrane was washed three times for 5 min in TBST. Membrane staining was visualized by incubation with a chemiluminescent HRP substrate. ERα, ERβ, ERRα, CD31, SOD2, and PGI_2_S were imaged using Luminata Forte (ECL; Merck Millipore, Darmstadt, Germany), and Flk-1 and eNOS were imaged using SuperSignal™ West Femto Maximum Sensitivity Substrate (FEMTO; Thermo Fisher Scientific, MA, USA). The images were digitalized using a ChemiDoc MP system (Bio-Rad, Hercules, CA, USA). Protein content was expressed as the mean of the duplicates and was presented in arbitrary units related to human standards normalized to the average of all samples loaded on the same gel.

**Table 1:** Overview of antibodies used for western blotting.

**Primary antibody**	**Function**	**Supplier**	**Catalog No**.	**Dilution of primary antibody**	**Blocking agent**	**Secondary antibody**	**Dilution of secondary antibody**	**Detection agent**
ERα	Receptor that binds oestrogenic compounds	Cell Sig.	8644	1:1,000	5% Milk, TBST	Goat Anti-Rabbit IgG	1:5,000	ECL
ERβ	Receptor that binds oestrogenic compounds	R&D Systems	MAP-7106	1:500	TBST	Goat Anti-Mouse IgG	1:5,000	ECL
ERRα	Estrogen-related receptor that can activate Estrogen Response Elements. Oestrogenic compounds can't be bound to ERRs	Abcam	76223	1:750	2% Milk, TBST	Goat Anti-Rabbit IgG	1:5,000	ECL
eNOS	Enzyme that is protective of the cardiovascular system, through the production of NO	Abcam	5589	1:10,00	5% Fish, TBST	Goat Anti-Rabbit IgG	1:5,000	FEMTO
PGI2S	Enzyme that produces prostacyclin, which is a vasodilatory compound and plays an important role in cardiovascular disease	Abcam	23668	1:300	3% BSA, TBST	Goat Anti-Rabbit IgG	1:5,000	ECL
SOD2	Transforms toxic superoxide, a product of the mitochondrial electron transport chain, to hydrogen peroxide	Merck Millipore	06-984	1:5,000	2% Milk, TBST	Goat Anti-Rabbit IgG	1:5,000	ECL
FLK1	Receptor with high affinity for VEGF, which mediates endothelial growth	Santa Cruz	393163	1:300	3% Fish, TBST	Goat Anti-Mouse IgG	1:5,000	ECL
CD31	Marker of endothelial cell differentiation	R&D Systems	AF806	1:250	2% Milk, TBST	Rabbit anti-sheep IgG	1:5,000	FEMTO

*ERα, estrogen receptor alpha; ERβ, estrogen receptor beta; ERRα, estrogen related-receptor alpha; eNOS, endothelial nitric oxide synthase; PGI2S, prostacylin synthase; SOD2, superoxide dismutase; FLK1, vascular endothelial growth factor receptor 2; CD31, cluster of differentiation 31; BSA, bovine serum albumin; TBST, Tris-buffered saline tween; ECL, Luminata Forte; FEMTO, SuperSignal™ West Femto Maximum Sensitivity Substrate*.

### Statistical Analysis

The statistical analyses were performed using RStudio (R-Studio, Version 4.0.0, R Foundation for Statistical Computing, Vienna, Austria). Graphs were made in GraphPad Prism (GraphPad, Version 8.4.3, San Diego, CA, USA). An a priori power calculation was made for the primary outcome; a change in plasma VCAM-1 levels, based on previous data from our laboratory (39). For a power of 0.8 and an alpha value of 0.05 *n* = 9 subjects were estimated to be required. Paired *t*-tests were used to compare the PLA and RC interventions. There was no observed order effect of the intervention (*p* = 0.994). Data are presented as mean ± SD. Individual values are shown in figures. Statistical significance was achieved if *p* < 0.05. Effect sizes (*d*) were calculated and reported for each parameter by dividing the mean of the differences by the standard deviation of the differences.

## Results

### Plasma Markers of Vascular Inflammation

**Figure 2:**
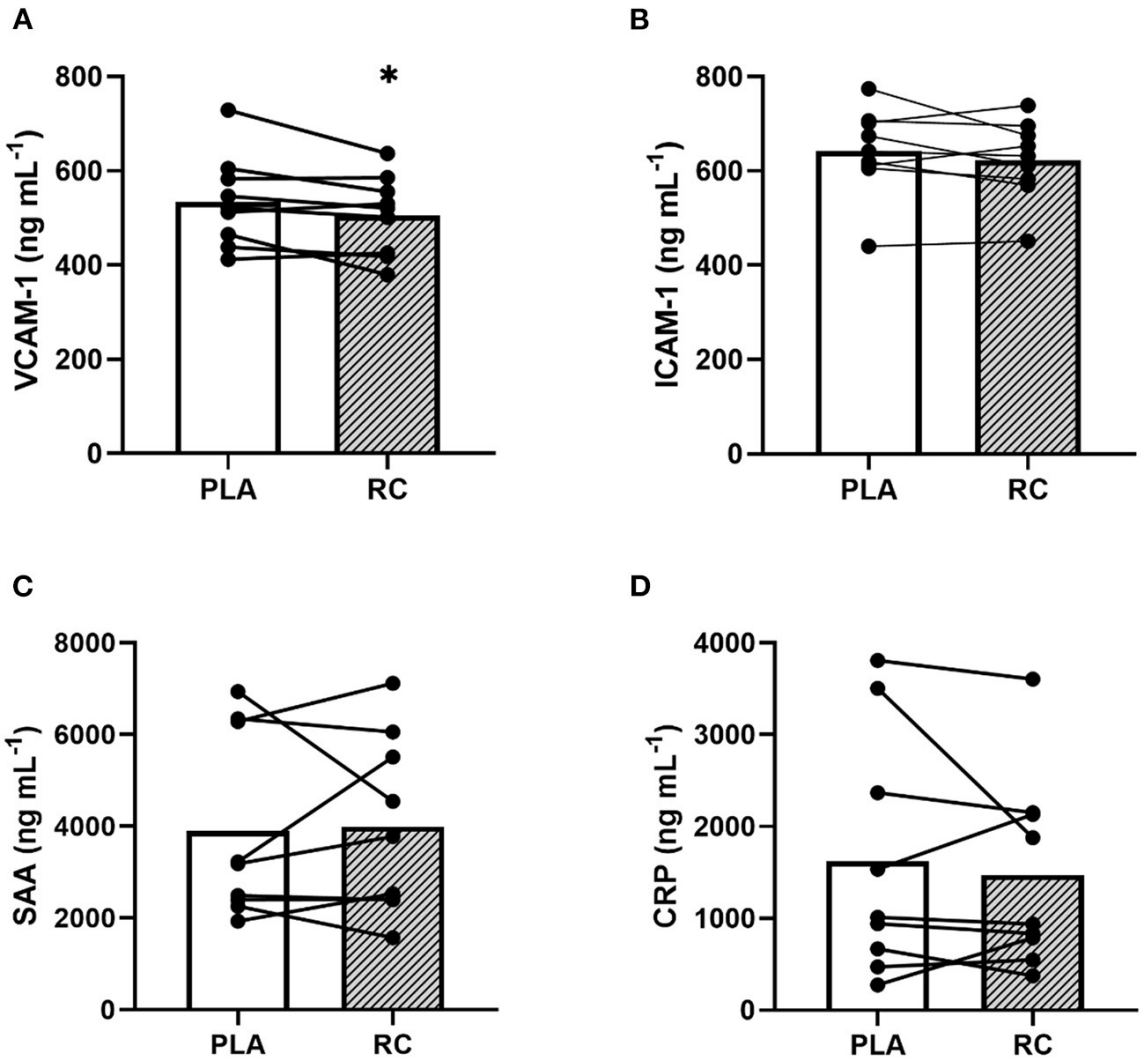
Levels of inflammatory markers in plasma samples obtained from early post-menopausal women following two weeks of twice daily supplementation with placebo (PLA) or fermented red clover extract (RC) (*n* = 9). **(A)** Vascular cell adhesion molecule 1 (VCAM-1), **(B)** intracellular adhesion molecule 1 (ICAM-1), **(C)** serum amyloid A (SAA), and **(D)** C reactive protein (CRP). *, indicates *p* < 0.05.

Plasma levels of VCAM-1 were significantly reduced by ~5.4% following RC compared to PLA (503.2 ± 84.4 vs. 534.8 ± 96.8 ng mL^−1^; *p* = 0.0321; *d* = 0.7; Figure 2A). However, there was no statistically significant effect of RC supplementation compared to PLA on ICAM-1 (628.2 ± 84.2 vs. 641.8 ± 93.2 ng mL^−1^; *p* = 0.1310; *d* = 0.4; Figure 2B), SAA (3,732.4 ± 1,927.6 vs. 3,894.2 ± 2,021.3 ng mL^−1^; *p* = 0.5877; *d* = −0.1; Figure 2C), or CRP (1,656.4 ± 1,354.4 vs. 1,620.5 ± 1,311.9 ng mL^−1^; *p* = 0.2536; *d* = 0.2; Figure 2D).

### Skeletal Muscle Estrogen and Estrogen Related-Receptor Content

**Figure 3:**
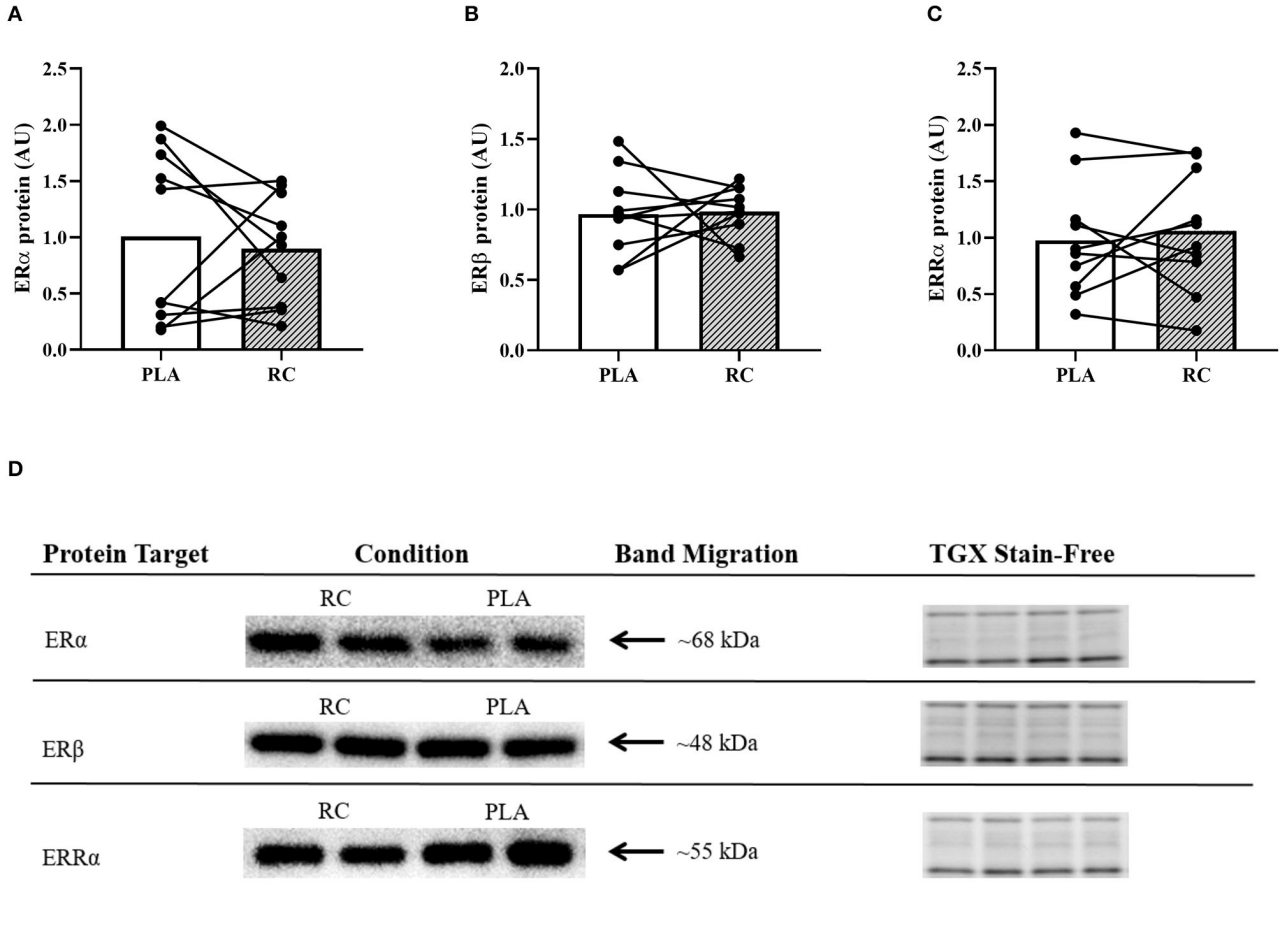
Protein expression of estrogen and estrogen-related receptors in skeletal muscle from early post-menopausal women following two weeks of twice daily supplementation with placebo (PLA) or fermented red clover extract (RC) (*n* = 10). **(A)** Estrogen receptor alpha (ERα), **(B)** Estrogen receptor beta (ERβ), and **(C)** Estrogen-related receptor alpha (ERRα). **(D)** Representative western blots and the corresponding TGX stain-free images.

The western blotting analysis showed no statistically significant effect of RC supplementation compared to PLA on ERα (0.89 ± 0.48 vs. 1.10 ± 0.76 AU; *p* = 0.3170; *d* = 0.2; Figure 3A), ERβ (0.99 ± 0.18 vs. 0.97 ± 0.30; *p* = 0.5545; *d* = 0.0; Figure 3B), and ERRα (0.95 ± 0.53 vs. 0.97 ± 0.51; *p* = 0.7025; *d* = −0.2; Figure 3C) protein content. Representive western blots are shown in (Figure 3D).

### Skeletal Muscle Protein Content of Enzymes

**Figure 4:**
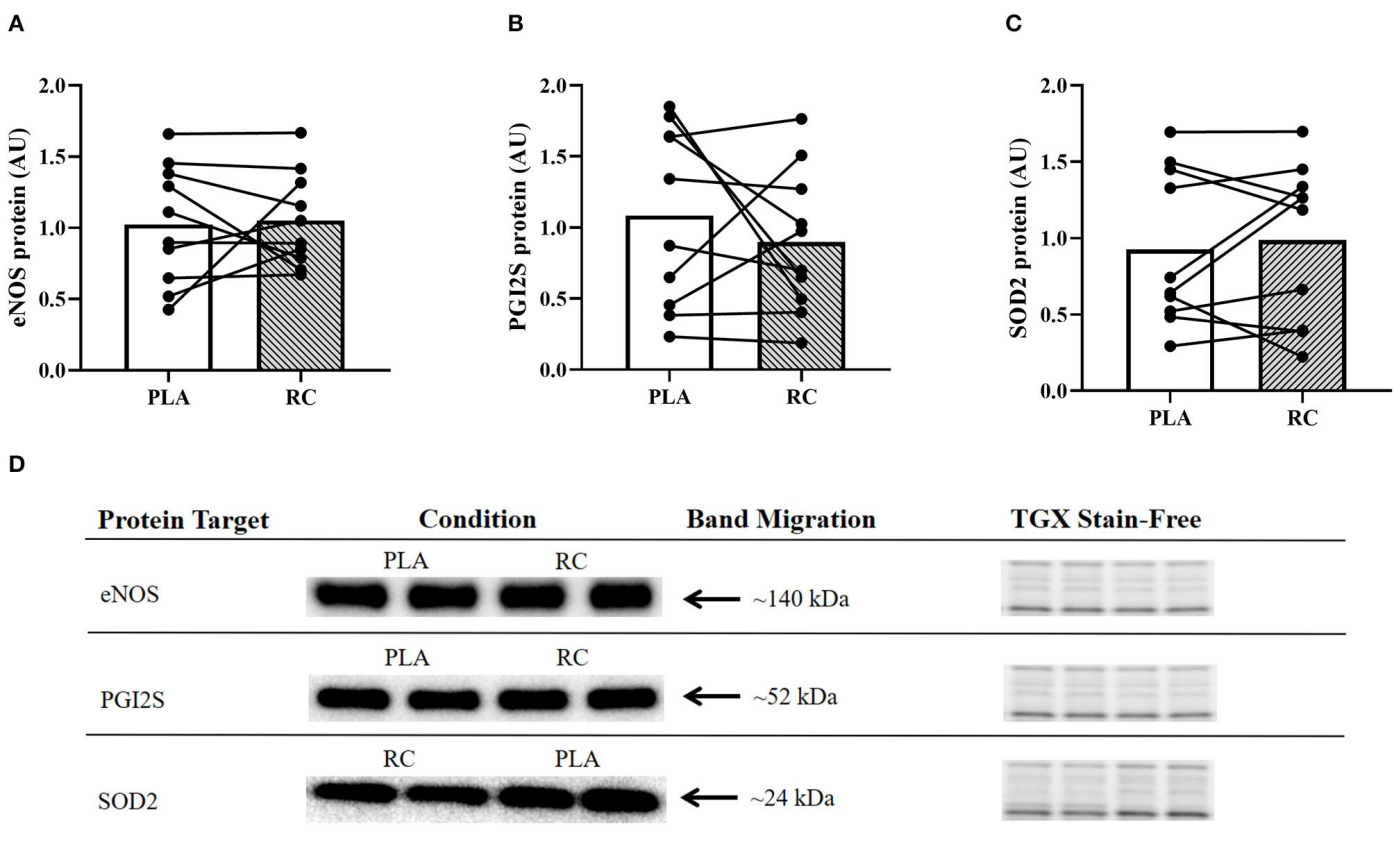
Expression of proteins associated with vasodilatory capacity in skeletal muscle from early post-menopausal women following 2 weeks of twice daily supplementation with placebo (PLA) or fermented red clover extract (RC) (*n* = 10). **(A)** Endothelial nitric oxide synthase (eNOS), **(B)** prostacyclin synthase (PGI_2_S), and **(C)** superoxide dismutase 2 (SOD2). **(D)** Representative western blots and the corresponding TGX stain-free images.

There was no statistically significant effect of RC supplementation compared to PLA on eNOS (1.05 ± 0.33 vs. 1.02 ± 0.42; *p* = 0.5837; *d* = −0.1; Figure 4A), PGI_2_S (0.90 ± 0.50 vs. 1.08 ± 0.63; *p* = 0.2056; *d* = 0.3; Figure 4B), and SOD2 (0.99 ± 0.52 vs. 0.93± 0.51; *p* = 0.7060; *d* = −0.2; Figure 4C) protein content. Representative western blots are shown in (Figure 4D).

### Skeletal Muscle Content of Angiogenic Proteins

**Figure 5:**
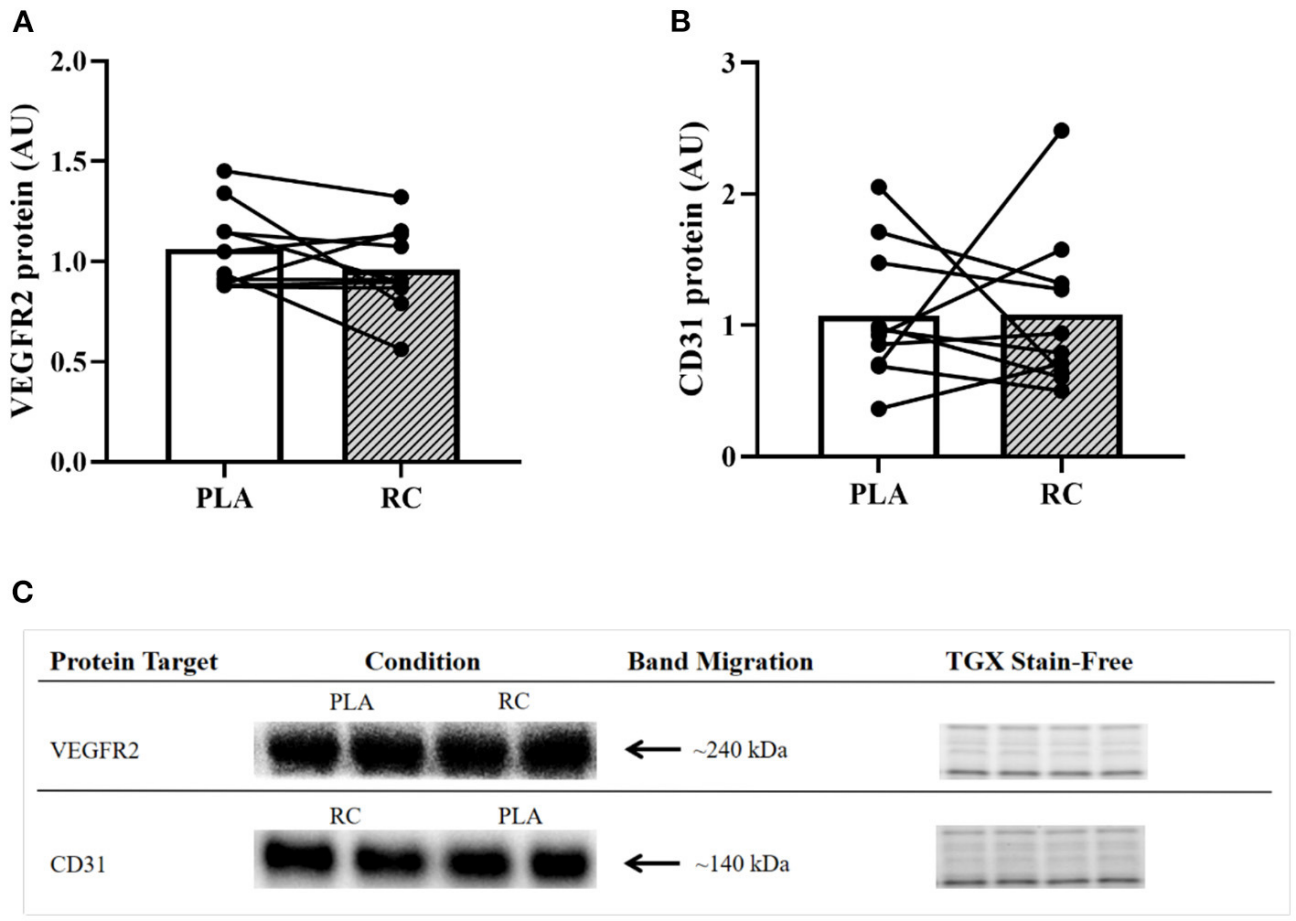
Expression of proteins associated with angiogenic potential in skeletal muscle from early post-menopausal women following 2 weeks of twice daily supplementation with placebo (PLA) or fermented red clover extract (RC) (*n* = 10). **(A)** Vascular endothelial growth factor receptor 2 (VEGFR2) and **(B)** cluster of differentiation 31 (CD31). **(C)** Representative western blots and the corresponding TGX stain-free images.

There was no statistically significant effect of RC supplementation compared to PLA on VEGFR2 (0.96 ± 0.22 vs. 1.06 ± 0.21; *p* = 0.1021; *d* = 0.4; Figure 5A) and CD31 (1.08 ± 0.61 vs. 1.07 ± 0.52; *p* = 0.5162; *d* = 0.0; Figure 5B) protein content. Representative western blots are shown in (Figure 5C).

## Discussion

The main objective of this study was to determine whether 2 weeks of twice daily supplementation with RC could improve markers of vascular health in the plasma and skeletal muscle of early post-menopausal women. The primary finding was a reduction in circulating VCAM-1 levels following 2 weeks of RC supplementation compared to PLA. RC supplementation did not lead to significant alterations in any of the other plasma markers of vascular inflammation or skeletal muscle protein content of estrogen and estrogen-related receptors as well as enzymes related to vascular function and angiogenesis.

### Plasma Markers of Vascular Inflammation

Vascular inflammation is characterized by the activation of cell adhesion molecules, which contributes to the development of atherosclerosis (40) and is inversely correlated with estrogen status (24, 41, 42). In this study, we measured plasma levels of VCAM-1, ICAM-1, SAA, and CRP as key markers of vascular inflammation and cell adhesion in response to short-term RC supplementation in early post-menopausal women. Corroborating our hypothesis, we found a statistically significant reduction (~5.4%), with a moderate-to-large effect size (*d* = 0.7), in the VCAM-1 level following short-term RC supplementation (Figure 2A). However, there were no statistically significant changes in CRP, SAA, or ICAM-1 levels (Figures 2B–D). Although, ICAM-1 had a small-to-moderate effect size (*d* = 0.4) and CRP had a small effect size (*d* = 0.2), suggesting possible reductions in these inflammatory markers following RC supplementation compared to PLA. A greater depth of research is required to validate these potential effects. In response to elevated circulating cytokines, VCAM-1 plays a crucial role in mediating immune cell adhesion to the vascular endothelium (40). Importantly, estrogen and phytoestrogens can elicit anti-inflammatory effects, thereby reducing vascular inflammation (24–26). We found that VCAM-1 was the only inflammatory marker that was significantly improved following RC supplementation. Interestingly, previous cell culture work has shown that estrogen elicits more robust beneficial effects on cytokine-stimulated VCAM-1 expression compared to other markers of vascular inflammation, such as ICAM-1 (43), which may explain the findings of the current study. In line with our findings, a previous investigation by (44) found a decline in plasma VCAM-1 in parallel with a reduction in arterial stiffness after 6 weeks of supplementation with isolated formononetin-rich isoflavones in healthy men and post-menopausal women. The lowering of VCAM-1 levels may indicate reduced atherosclerosis, as the level of circulating cell adhesion molecules is positively correlated with the degree of atherosclerosis in humans (45). The mechanisms underpinning the statistically significant reduction in VCAM-1 levels remains to be elucidated. Specifically, it is unclear whether isoflavones elicit a direct anti-inflammatory effect or if they facilitate anti-inflammatory signaling cascades, such as the promotion of NO or PGI_2_ release from the vascular endothelium.

### Regulation of Skeletal Muscle Estrogen Receptors and Microvascular Proteins

A depth of research has emerged demonstrating the importance of the estrogen receptor-mediated pathways for cardiovascular and metabolic health (46, 47). Notably, in post-menopausal women, the impaired interaction of estrogen with its receptors in the skeletal muscle microvasculature may play a vital role in the development of cardiovascular and metabolic diseases. In this study, we sought to determine whether short-term RC supplementation could mitigate some of these impairments.

#### Estrogen Receptor Content

A large proportion of the beneficial effects of estrogen are mediated by the activation of signaling cascades when estrogen binds to its steroid receptors (48). Recent research has shown that menopause is associated with a decline in skeletal muscle estrogen receptor content (49) and the tendency toward a higher ratio of ERβ to ERα (50) Together, these factors have been linked to adverse cardiovascular outcomes (49). Importantly, in rodent models of menopause, estrogen receptor content can be increased in response to recurring stimuli that utilize these pathways, such as hormone replacement therapy (51) and endurance exercise training (52). Moreover, the ratio of ERβ to ERα can be restored to pre-menopausal levels following treatment with estrogen (53). However, in the current study, we did not observe a change in the expression of estrogen receptor content following RC supplementation as assessed by ERα and ERβ (Figures 3A,B). Oxfeldt et al. (36) also found no statistically significant change in ERβ protein content in skeletal muscle following RC supplementation compared to PLA. Importantly, ERβ protein content was determined using two different antibodies (Table 1), highlighting the reproducibility of our findings.

#### Protein Content of Enzymes, Angiogenic Markers, and ERRα

Previous studies have shown that within the first few years after menopause women have reduced vascular function (54) and angiogenic potential (12). Although exercise training interventions have been shown to enhance markers of vascular function (55) and angiogenesis (56) in post-menopausal women, no study to date has explored the effects of phytoestrogen supplementation on such markers. To investigate the effects of RC supplementation on skeletal muscle enzymes, we measured eNOS and PGI_2_S, which are responsible for producing the vasodilators NO and PGI_2_, respectively (57). Additionally, we measured SOD2 as it is an antioxidant enzyme that can facilitate NO bioavailability (58). Contrary to our initial hypothesis, we did not find any statistically significant changes in the protein content of these skeletal muscle enzymes following RC supplementation compared to PLA (Figures 4A–C). However, following RC supplementation compared to PLA, we observed small effect sizes for PGI_2_S (*d* = 0.3) and SOD2 (*d* = −0.2), suggesting a potential decrease and increase in protein content, respectively. More research is required to validate and draw conclusions regarding these potential effects. Our results are in contrast with recent findings in human endothelial cells, which demonstrated that red clover elicits increased eNOS expression, enhanced NO production, and lowered reactive oxygen species production (59). Although we did not observe a statistically significant increase in eNOS or PGI_2_S protein expression, it is possible that RC supplementation improved the activity of these enzymes and thereby, contributed to the observed reduction in vascular inflammation in this study (24, 60). Evidently, future work is required to tease apart these mechanisms. Moreover, VEGFR2 and CD31 were measured to assess the effects of RC supplementation on angiogenic markers in skeletal muscle. We did not observe any statistically significant changes in angiogenic proteins following RC supplementation compared to PLA (Figures 5A,B). However, we did observe a small-to-moderate effect on VEGFR2 protein content (*d* = 0.4), suggesting a possible decrease following RC supplementation compared to PLA. A greater body of evidence is required to support or refute this potential effect. Lastly, we measured ERRα protein content, as it is a non-steroid receptor that is abundant in skeletal muscle and exclusively expressed in endothelial cells (61). Importantly, the isoflavones in RC have been shown to behave as agonists that promote ERRα activity (62), which could potentially increase eNOS expression (63) as well as angiogenesis (61). However, we did not see any change in ERRα protein content following RC supplementation (Figure 3C).

### Study Limitations

Ten subjects were included in the cross over design and it cannot be excluded that the power was too low for some of the variables measured. In addition, The two-week RC intervention may have been too short to elicit an optimal effect on the plasma inflammatory profile and skeletal muscle proteins. However, our intervention period was selected based on the premise that as little as one week of oestrogen therapy has been shown to induce significant changes in skeletal muscle protein content (64). To limit the invasive procedures for the participants, plasma and skeletal muscle samples were only obtained at the end of the 14-day interventions and, therefore, we do not have baseline measurements. However, the study was of a randomized, crossover design and we did not observe an order effect, which minimizes the likelihood of this limitation. A levels *via* high performance liquid chromatography (65). Therefore, it is unclear how much of the RC was successfully delivered to the skeletal muscle. Lastly, the study participants, though sedentary, were healthy and since this study did not include a functional vascular measure, it is possible that the vascular function status of the participants may have been too high to see beneficial effects with RC supplementation.

### Future Directions

The findings from this study have unearthed novel evidence regarding the cardiovascular benefits of RC, which support the known therapeutic potential of RC in relieving menopausal symptoms (66), promoting bone health (33), and minimizing skeletal muscle breakdown (36) in post-menopausal women. Future investigations are required to elucidate the optimal dosing regime and mechanisms of action, together with reporting of potential side effects. Importantly, future investigations should consider increasing the duration of supplementation to optimize the therapeutic effects on cardiovascular and potentially metabolic health. Moreover, future investigations should consider combining RC supplementation with exercise training to determine whether this strategy can be used to maximize cardiovascular and metabolic benefits in post-menopausal women. Lastly, the optimal timing after menopause for initiation of RC supplementation should be evaluated, as a growing body of evidence suggests therapeutic interventions (e.g., HRT or aerobic exercise training) are more successful in early post-menopausal women compared to late post-menopausal women (67).

### Conclusions

This study provides the first evidence that as little as 2 weeks of twice-daily supplementation with RC reduces VCAM-1 expression and significantly improves vascular inflammation in early post-menopausal women.

## Data Availability Statement

The raw data supporting the conclusions of this article will be made available by the authors, without undue reservation.

## Ethics Statement

The studies involving human participants were reviewed and approved by Central Denmark Region Committees on Health Research Ethics (1-10-72-212-19). The participants provided their written informed consent to participate in this study.

## Author Contributions

KW, LN, MO, MH, and YH conceived the study design. KW, LN, and YH designed the experiments. KW, LN, and MO collected the data. KW and LN reduced and synthesized the data and drafted the manuscript. KW, LN, LG, SC, and YH interpreted the data. All authors critically revised and approved the final version of the manuscript.

## Funding

This study was supported by Frimodt-Heineke Fonden, Læge Sofus Carl Emil Friis og Hustru Olga Doris Friis' Legat, Aarhus University Research Foundation (AUFF), and the Toyota Foundation, Denmark. KW is supported by a CGS-D scholarship through the National Sciences and Engineering Research Council (NSERC) of Canada. LN is supported by a grant from the Nordea-fonden.

## Conflict of Interest

The authors declare that the research was conducted in the absence of any commercial or financial relationships that could be construed as a potential conflict of interest. The reviewer PJ declared a shared affiliation with several of the authors MH and MO, to the handling editor at time of review.

## Publisher's Note

All claims expressed in this article are solely those of the authors and do not necessarily represent those of their affiliated organizations, or those of the publisher, the editors and the reviewers. Any product that may be evaluated in this article, or claim that may be made by its manufacturer, is not guaranteed or endorsed by the publisher.

## Abbreviations

BCA: Bicinchoninic Acid
CRP: C-Reactive Protein
CD31: Cluster of Differentiation 31
EDTA: Ethylenediaminetetraacetic Acid
eNOS: Endothelial Nitric Oxide Synthase
RC: Fermented Red Clover Extract
FLK1: Fetal Liver Kinase 1
HRT: Hormone Replacement Therapy
HRP: Horseradish Peroxidase
ICAM-1: Intracellular Adhesion Molecule 1
ERα: Estrogen Receptor Alpha
ERRα: Estrogen-Related Receptor Alpha
ERβ: Estrogen Receptor Beta
PLA: Placebo
PGI_2_: Prostacyclin
PGI_2_S: Prostacyclin Synthase
PVDF: Polyvinylidene Difluoride
rpm: Revolutions Per Minute
SAA: Serum Amyloid A
SD: Standard Deviation
SOD2: Superoxide Dismutase 2
TBST: Tris-Buffered Saline Tween
TGX: Tris-Glycine eXtended
VCAM-1: Vascular Cell Adhesion Molecule 1
VEGFR2: Vascular Endothelial Growth Factor Receptor 2.
